# Exendin-4-enriched exosomes from hUCMSCs alleviate diabetic nephropathy via gut microbiota and immune modulation

**DOI:** 10.3389/fmicb.2024.1399632

**Published:** 2024-08-30

**Authors:** Liping Wang, Aihua Liang, Jukai Huang

**Affiliations:** ^1^Key Laboratory of Beijing for Identification and Safety Evaluation of Chinese Medicine, Institute of Chinese Materia Medica, China Academy of Chinese Medical, Beijing, China; ^2^Department of Endocrinology, Beijing University of Chinese Medicine, Dongzhimen Hospital, Beijing, China

**Keywords:** diabetic nephropathy, kidney injury, human umbilical cord mesenchymal stem cells, CD4 + regulatory T cells, gut microbiota metabolism, *Prevotella*

## Abstract

**Introduction:**

Diabetic nephropathy (DN) presents a significant therapeutic challenge, compounded by complex pathophysiological mechanisms. Recent studies suggest Exendin-4 (Ex-4) as a potential ameliorative agent for DN, albeit with unclear mechanisms. This research investigates the effects and underlying mechanisms of Ex-4-enriched exosomes derived from human umbilical cord mesenchymal stem cells (hUCMSCs) on DN, focusing on their renoprotective properties and interactions with gut microbiota.

**Method:**

Exosomes from hUCMSCs (hUCMSCs-Exo) were loaded with Ex-4 via electroporation. A streptozotocin (STZ) -induced DN mouse model was employed to assess the therapeutic impact of these engineered exosomes. The study further explored immune cell dynamics, mainly CD4^+^ regulatory T (Treg) cells, using bioinformatics, flow cytometry, and the influence of gut microbiota through antibiotic treatment and specific bacterial reintroduction.

**Results:**

Treatment with hUCMSCs-Exo@Ex-4 significantly improved key DN markers, including blood glucose and proteinuria, alleviating kidney damage. A notable decrease in natural Treg cell infiltration in DN was observed, while Ex-4-loaded exosomes promoted CD4^+^ Treg cell induction. The therapeutic benefits of hUCMSCs-Exo@Ex-4 were diminished upon CD4^+^ Treg cell depletion, underscoring their role in this context. Notably, CD4^+^ Treg cell induction correlated with the presence of *Prevotella* species, and disruption of gut microbiota adversely affected these cells and the therapeutic efficacy of the treatment. However, the reintroduction of *Prevotella* strains counteracted these adverse effects.

**Discussion:**

This study elucidates a novel therapeutic mechanism of Ex-4-loaded hUCMSCs exosomes in DN, highlighting the induction of CD4^+^ Treg cells mediated by specific gut microbiota components. These findings underscore the potential of leveraging gut microbiota and immune cell interplay in developing effective DN treatments.

## Introduction

Diabetic nephropathy (DN), as a severe complication of diabetes mellitus (DM), is related to elevated morbidity and mortality in patients with DM (Tervaert et al., 2010; Umanath and Lewis, 2018). DN, one of the most prevalent chronic kidney diseases, is the main reason for end-stage renal disease in adults (Alicic et al., 2017), associated with continuous proteinuria and reduction in renal functions (Selby and Taal, 2020). The pathogenesis of DN remains unclear, and the existing treatments have not achieved satisfactory effects (Tang et al., 2020), which calls for novel therapeutic strategies.

In recent years, mesenchymal stem cells (MSCs) have been found in tissues, including the umbilical cord, which has been applied in treating human diseases due to their self-renewal ability and multi-differentiation potential (Wang et al., 2021). The roles of human umbilical cord-derived mesenchymal stem cells (hUCMSCs) in DN have also been explored (Xiang et al., 2020). The therapeutic effect of MSCs largely depends on the released factors, such as exosomes (Ebrahim et al., 2018). Exosomes, a significant type of extracellular vesicles (EVs), are secreted by various cells (Isaac et al., 2021). Exosomes can be transported to distant cells to regulate intercellular communication in health and disease through cargo RNAs and proteins (Hao et al., 2021). Recent evidence has also demonstrated that exosomes affect pathophysiological processes in DN (Chen J. et al., 2021), and exosomes from human umbilical cord MSCs alleviate type 2 DM (T2DM) by negating peripheral insulin resistance and repressing β-cell destruction (Sun et al., 2018). Extracellular vesicles, known for their ability to carry various drugs and unique biological characteristics, have gained significant attention as a novel drug delivery vehicle (Kalluri and LeBleu, 2020). Drug delivery systems based on extracellular vesicles have been extensively studied, showing promising clinical applications, such as using exosomes loaded with curcumin for the treatment of encephalitis and exosomes loaded with catalase for the treatment of Parkinson’s disease (Zhuang et al., 2011; Haney et al., 2015).

Additionally, regulatory T (Treg) cells are implicated in the pathogenesis of DN (Rayego-Mateos et al., 2020). Treg cells, a specialized subset of T cells, play a crucial role in modulating inflammatory responses and maintaining immune homeostasis, particularly in the treatment of inflammatory diseases such as DN (Eller et al., 2011; Zhang et al., 2020). Moreover, the gut microbiota is associated with the onset and progression of DN. The gut microbiota, comprising microbes like archaebacteria and bacteria (Chen W. et al., 2021), exhibits characteristic alterations in bacterial diversity and composition in patients with DN, with a notable absence of species like *Escherichia*, *Citrobacter*, *Klebsiella*, and *Roseburia* (Wang et al., 2022). This suggests that dysbiosis in the gut microbiota of diabetic patients significantly influences the development and progression of the disease. The gut microbiota contributes to the advancement of diabetes and its renal complications by lowering glucose tolerance and promoting insulin resistance (Zaky et al., 2021). Furthermore, the gut microbiota participates in the progression of DN through inflammatory mediation (Cai et al., 2022). Notably, *Prevotella copri* in the gut microbiota elevates branched-chain amino acid levels in the peripheral circulation, inducing insulin resistance, leading to glucose intolerance, and facilitating the development of DN (Pedersen et al., 2016). Additionally, there is an association between Treg cells and the gut microbiota (Liu et al., 2021). The gut microbiota contributes to the communication between intestinal microbes and the immune system by fermenting and producing short-chain fatty acids, thereby enhancing the induction and differentiation of Treg cells (Arpaia et al., 2013). Hence, Treg cells and the gut microbiota may represent novel therapeutic targets for the management of DN.

In this study, we established STZ-induced DN rat models, and Ex-4 was delivered into exosomes derived from hUCMSCs by electroporation to explore the therapeutic effect and underlying mechanisms of hUCMSCs-Exo@Ex-4 in DN, which may be associated with CD4^+^ Treg cell immune infiltration and gut microbiota metabolism.

## Materials and methods

### Experimental animals and cells

Wild-type C57BL/6 mice (aged 5 ~ 6 weeks) were purchased from Beijing Huafu Kang Biotechnology Co., Ltd. (Beijing, China). Mice were raised at 22 ~ 25°C with humidity of 60% ~ 65% under a 12-h light and dark cycle, with free access to sterilized food and water. The experiment was conducted after acclimatization for 1 week. This study received approval from the Animal Care and Use Committee of Viewsolid Biotech Co., Ltd. (approval no. VS2126A01208).

hUCMSCs (HUM-iCell-e009, iCell Bioscience Co., Ltd., Shanghai, China) were cultured in serum-free human MSC medium (CM-SC01, Procell Life Science & Technology Co., Ltd., Wuhan, China) supplemented with 100 U/mL penicillin and 100 μg/mL streptomycin (10,378,016, Invitrogen, Carlsbad, California), which was placed in an incubator with 5% CO_2_ at 37°C.

### Isolation and characterization of exosomes from hUCMSCs

Exosomes were isolated using the Ribo™ exosome isolation reagent (C10130-1, Guangzhou RiboBio Co., Ltd., Guangzhou, China). After incubation for 72 h, the medium supernatant was collected and centrifuged at 2,000 × g at 4°C for 30 min to remove the floating cells, cell debris, and unwanted proteins. The cell supernatant was transferred to a new tube and placed on ice. Next, a third volume of Ribo™ exosome isolation reagent was added into the tube, shaken, and mixed thoroughly. Then, the samples were placed in a 4°C refrigerator overnight. The next day, the 2 mL mixture was pipetted to a centrifuge tube, followed by centrifugation at 1,500 × g at 4°C for 30 min to remove the supernatant and retain the precipitation. This centrifugation step was repeated thrice until all samples were transferred. The exosomes were eluted, and the precipitation was resuspended in 1 × PBS.

The morphology of exosomes was observed and photographed under a transmission electron microscope (TEM) (Talos F200X S/TEM, Opton, Beijing, China). Subsequently, the protein expression of exosome surface markers (CD63, HSP70, and Calnexin) was analyzed using Western blot analysis. The diameter of exosomes was detected by dynamic light scattering using the Zetasizer Nano-ZS90 instrument (Malvern, United Kingdom).

### Western blot

The exosomes were dissolved in radioimmunoprecipitation assay (RIPA) buffer (AR0102, Wuhan Boster Biological Technology Co., LTD., Wuhan, China) for lysis, and the total protein concentration was determined using the bicinchoninic acid (BCA) assay kit (AR0146, BOSTER). Proteins (30 μg) were separated by 10% sodium dodecyl sulfate-polyacrylamide gel electrophoresis (SDS-PAGE) and electro-transferred to polyvinylidene fluoride (PVDF) membranes (IPFL00010, Merck Millipore, Billerica, MA). The membranes were blocked in the 5% bovine serum albumin (BSA) at room temperature for 2 h to block the nonspecific binding and then probed with primary antibodies, CD63 (ab216130, 1:1000, Abcam, Cambridge, United Kingdom), HSP70 (ab2787, 1:1000, Abcam), and Calnexin (ab22595, 1:1000, Abcam) at 4°C overnight. Next, after washing with Tris Buffered Saline with Tween (TBST), the membrane was added with horseradish peroxidase (HRP)-labeled goat anti-rabbit immunoglobulin G (IgG; ab97051, 1:10,000) at room temperature for 1 h. After washing, the membrane was added with the enhanced chemiluminescence (ECL) working solution (WBKLS0050, Millipore).

### Loading of Ex-4 by hUCMSCs-Exo through electroporation

Exendin-4 (HY-13443, MedChemExpress) was delivered onto the hUCMSCs-Exo (total protein concentration of 10 μg) using neon electroporation (1,000 V, 10 ms, 2 pulses). Before electroporation, the 10 μg Ex-4 was mixed into the R buffer of the Neon kit (MPK10096, Invitrogen). After electroporation, the exosomes were washed in PBS twice and centrifuged at 100,000 × g. To calculate the loading efficiency, the samples were diluted with 100 × PBS and centrifuged at 100,000 g for 70 min to remove the free Ex-4. The loading rate of Ex-4 was measured using a spectrophotometer (942,350,033,312, Thermo Fisher Scientific, Waltham, MA).

Exo@Ex-4 (50 mg) was sonicated in 1 mol/L HCl (50 mL) for 1 h. The concentration of Ex-4 in the supernatant was measured using a 265 nm UV–visible spectrophotometer (UV–VIS 8500, Techcomp, Beijing, China), with the exosome supernatants not transfected with Ex-4 as a control. The loading capacity of Exo@Ex-4 was calculated by the following formulas: LC% = (W1/W2) × 100%, where W1 is the weight of Ex-4 encapsulated in the exosomes, W2 is the gross weight of the Exo@Ex-4. Under the optimal electroporation conditions, the HPLC result indicated that the loading capacity of Exo@Ex-4 was 3.1%. The Ex-4 content was confirmed using an ELISA kit (ab106796, Abcam).

### Enzyme-linked immunosorbent assay

ELISA detected the Ex-4 content in hUCMSCs-Exo@Ex-4. The pre-coated Ex-4 antibody (ab106796, Abcam) was coated in the microwells, followed by adding with hUCMSCs-Exo@Ex-4, standard, and HRP-labeled detection antibody simultaneously after incubation and washing. The samples were developed with the substrate TMB. Finally, the absorbance (OD value) was measured using the Microplate Reader at the wavelength of 450 nm.

### Construction of DN mouse models

The mice were intraperitoneally injected with STZ (Aladdin, Shanghai, China) at a dose of 50 mg/kg for 7 consecutive days to induce DN mouse models, while the control mice were injected with the sodium citrate buffer with the same volume and same pH of STZ. Mice with fast blood glucose levels over 16.7 mM were confirmed as diabetes. The urine was collected from mice using the metabolic cages to measure urinary protein content from 4 to 6 weeks after STZ treatment. The 24-h urinary protein ≥ 30 mg/24 h was diagnosed as DN (Xiang et al., 2020).

The successful DN mouse model received multiple injections of extracellular vesicles every 2 weeks between 6 and 12 weeks of age (Zhang et al., 2021). Mice were randomly divided into groups and injected with hUCMSCs-Exo or hUCMSCs-Exo@Ex-4 via the tail vein. A volume of 200 μL per injection was administered into the lateral tail vein using a 27-gage needle. The injection position was at the midpoint of the tail to ensure proper administration.

To observe the effect of hUCMSCs-Exo and hUCMSCs-Exo@Ex-4 on kidney injury in DN mice, the successful DN mouse models were injected with exosomes from 6 to 12 weeks. Mice were randomly divided into the following groups: control group (control mice were intraperitoneally injected with PBS), DN (untreated DN mouse models), DN + hUCMSCs-Exo (DN mice were injected with 10 μg hUCMSCs-Exo via tail vein), and DN + hUCMSCs-Exo@Ex-4 (DN mice were injected with 10 μg hUCMSCs-Exo@Ex-4 via tail vein) group.

To consume CD4^+^ Treg cells *in vivo*, mice were injected with 500 μg anti-CD25 antibody (13–0252-82, Invitrogen) on days 1 and 3 before STZ administration and on days 7 and 14 days after STZ administration to exhaust CD4^+^ Treg cells. The CD25 antibody homologous IgG (ab280756, Abcam) served as a control antibody. DN mice were divided into the DN + hUCMSCs-Exo + control Ab, DN + hUCMSCs-Exo + CD25Ab, DN + hUCMSCs-Exo@Ex-4 + control Ab, and DN + hUCMSCs-Exo@Ex-4 + CD25Ab groups.

To observe the role of intestinal microflora in hUCMSCs@Ex-4-Exo in DN mice, DN mice were treated with the following combination of antibiotics (ABX) (stopped at the end of week 6 to week 12). Ampicillin (AMP, 1 g/L, A9518), metronidazole (1 g/L, PHR1052), vancomycin (500 mg/L, V2002) and neomycin (1 g/L, N6386) or AMP alone were added in drinking water for mice. Short-chain fatty acids (SCFAs) treatment: 100 mM sodium acetate (32319), 50 mM sodium butyrate (B5887), and 100 mM sodium propionate (P5436) were dissolved in drinking water for mice. The above ABX were all purchased from Sigma-Aldrich. Prevotella Inoculation: *Prevotella copri* (ATCC® 33547™) and *Prevotella melaninogenica* (ATCC® 25845™) were cultured anaerobically at 37°C on liquid thioglycolate medium for 3 days prior to use.^1^

After centrifugation, the bacteria were suspended in a liquid medium, followed by treatment with 200 μL inoculum (at 1 × 10^8^) once every 2 days for 4 weeks. One hour before bacterial gavage, Mice were intraperitoneally injected with 3 mg cimetidine hydrochloride (PHR1089, Sigma-Aldrich, St Louis MO) in 100 μL PBS to inhibit gastric acid secretion and improve colonization. The treatment with SCFAs (at a concentration of 2 mM) and Prevotella were administered 2 days after the completion of the antibiotic treatment. DN mice were divided into the DN + hUCMSCs-Exo@Ex-4 + ABX, DN + hUCMSCs-Exo@Ex-4 + AMP, DN + hUCMSCs-Exo@Ex-4 + ABX + BM (blank medium), DN + hUCMSCs-Exo@Ex-4 + ABX + PM, DN + hUCMSCs-Exo@Ex-4 + ABX + PC, and DN + hUCMSCs-Exo@Ex-4 + ABX + SCFAs groups.

There were 12 mice per group. Body weight and blood glucose concentrations were measured every 2 weeks during the experiment. Urine samples were used to detect 24-h urinary albumin and urinary albumin/creatinine ratio (UACR). All mice were euthanized after 12 weeks and weighed, followed by a dissection of the renal tissues. The renal tissues were weighed to calculate the ratio of renal tissues to body weight.

### Plasma biochemical analysis

Blood samples were collected from the ophthalmic venous for biochemical analysis. The plasma insulin levels were measured according to the instructions of a commercial mice insulin ELISA kit (EMINSX5, Invitrogen).

### Measurement of urinary protein and UACR at 24 h

The mice were placed in a metabolic cage. Urine was collected at any time point to measure urinary albumin and creatinine using mouse albumin-specific ELISA (Albuwell M kit) and creatinine detection kit (ab 65,340, Abcam). The UACR was calculated using the following formula: UACR (mg/g) = urinary microalbumin (mg/L) ÷ urinary creatinine (g/L).

### RNA extraction and quantification

TRIzol (16,096,020, Thermo Fisher Scientific) was used to extract total RNA. Next, the total RNA was reversely transcribed into cDNA using the cDNA first strand synthesis Kit (D7168L, Beyotime Institute of Biotechnology, Shanghai, China). Reverse transcription-quantitative polymerase chain reaction (RT-qPCR) was conducted using the RT-qPCR kit (Q511-02, Vazyme Biotec, Nanjing, China). The PCR amplification was performed using the Bio-rad real-time quantitative PCR instrument CFX96. The primer sequences of genes (Supplementary Table S1) were designed and provided by Sangon Biotechnology Co. Ltd. (Shanghai, China). The 2^-ΔΔCt^ method was used to quantify relative expression levels of target genes with the glyceraldehyde-3-phosphate dehydrogenase (GAPDH) as an internal reference.

In 16S rRNA qRT-PCR, fecal samples were collected using polyester swabs and air-dried at room temperature for at least 24 h. DNA extraction was conducted using the QIAamp® DNA Mini Kit (Qiagen, Valencia, CA) following the manufacturer’s “Isolation of Total DNA from Body Fluid Stains” protocol. After incubation at 70°C for 10 min, the swabs were placed in a DNA IQ™ Spin Basket (Promega, Madison, WI) to collect residual liquid, returned to the lysis tube, and centrifuged at 3350 g for 5 min. Reagent blanks were included in the extraction to assess contamination. All samples and reagent blanks were eluted in 50 μL of ATE buffer (Qiagen) and quantified for total DNA using NanoDrop™ 2000 (Thermo Fisher Scientific) before storage at −80°C.

gBlocks® gene fragments (IDT) were utilized as qPCR standards with the sequence: AGGCAGGCGG AATTCGTGGT GTAGCGGT GAAA TGCTTAGATAT CACGAAGAACTCCGATTGCGA AGGC AGCTTGCTGGACT GTAACTGACGCTGATGCT CGAAAGT GTGGGTATCAAACAGGA TTAGATACCCT GGTAGTCCA CACAGTA AACGATGAATACTC GCTGTTTGCGATATAC. The gBlocks® were reconstituted in TE buffer at 10 ng/μl, incubated at 50°C for 20 min. Dilution series ranging from 5 pg./μl to 0.05 fg/μl were prepared and stored at −20°C. The qPCR reaction mixture comprised 5 μL of PrimeTime® Gene Expression Master Mix (2X) (IDT), 2.5 μL of nuclease-free water, 0.5 μL of 20X primer mix, and 2 μL of the sample, totaling 10 μL. Primer concentration was 400 nM, and probe concentration was 200 nM. The thermal cycling parameters were 95°C for 3 min, followed by 40 cycles of rapid cycling (95°C for 5 s, 60°C for 30 s).

Data analysis was performed using R version 4.1.1 (R Foundation, Vienna, Austria). The data was randomly divided into training and validation sets (70 and 30%, respectively) using the sample() function. In each iteration, a classification and regression tree (CART) model was created using the rpart package, repeated 15 times. Confusion matrices and classification accuracy percentages were reported based on one iteration of the validation dataset. Where applicable, all data were presented as means with standard deviations (Lewis and Seashols-Williams, 2022).

### Hematoxylin and eosin staining

The renal tissues of mice were collected and fixed in 10% neutral formalin. Next, the paraffin-embedded tissues were sliced into sections dewaxed with xylene. Sections were stained with hematoxylin and then eosin and observed under an optical microscope.

### Periodic acid Schiff staining

PAS staining was conducted to measure the mesangial matrix expansion. The sections were soaked in a high-iodized acid alcohol solution for 10 min, washed with 70% alcohol, immersed in a reducing solution for 1 min, and then washed with 70% alcohol. Subsequently, the sections were placed in a colorless salt-based magenta solution for 1–1.5 h. The nuclei were counterstained with hematoxylin for 3–5 min and observed under a microscope.

### Immunohistochemical staining

The pancreatic tissues or renal tissue sections were immersed in 3% H_2_O_2_ at room temperature for 10 min to block endogenous peroxidase. The sections were incubated with citrate acid buffer and boiled in the microwave oven for antigen retrieval. After incubation with normal goat serum blocking solution, the sections were probed with diluted primary antibodies, insulin antibodies (ab181547, 1:100, Abcam), WT-1 (ab 180,840, 1:100, Abcam), CD4 (ab 183,685, 1:500, Abcam), and CD8 (ab 217,344, 1:1000, Abcam) at 4°C overnight. The sections were incubated with the secondary antibody goat anti-rabbit IgG (ab6721, 1:500, Abcam) for 30 min. After development using the DAB kit (Sigma-Aldrich), the sections were stained with hematoxylin and observed under an upright microscope (BX63, Olympus, Tokyo, Japan). The insulin-positive regions were detected with the insulin immunostaining method using an image analysis system (Aperio Scanscope System, Vista, CA).

### Masson trichrome staining

The paraffin-embedded sections of renal tissues were immersed in 10% trichloroacetic acid and 10% potassium dichromate liquid for 40 min, respectively. After hematoxylin staining, the sections were immersed in 1% ponceau (P0012, Solarbio) and 1% magenta mixture (G0031, Solarbio) for 40 min and treated with 1% glacial acetic acid (first) and 1% molybdate acid (later) to terminate the reaction. Sections were observed and photographed under the upright microscope (BX63, Olympus). Five visual fields were randomly selected for each section.

### Bioinformatics analysis

The DN-related microarray data (GSE142153) was downloaded from the Gene Expression Omnibus (GEO) database, which included 10 normal human (Normal) samples and 23 peripheral blood samples of DN patients. Differential gene analysis was performed using the R software “limma” package with |logFC| > 1 and *p* < 0.05 to select the differentially expressed genes (DEGs) in the microarray data GSE114868. Volcano maps were drawn using the R software “ggplot2” package, and the heat map of DEGs was drawn using the R software “heatmap” package. The R software “clusterprofiler” package was utilized for functional enrichment analysis of the screened target genes. The Fisher test was employed to identify significantly enriched KEGG pathways. *p* < 0.05 was considered statistically significant.

The ImmuCellAI algorithm estimated infiltrating immune cells in the DN samples. The abundance of immune cells in all samples was measured with normalized gene matrices using the ImmuCellAI online analysis. The results were visualized by the “pheatmap” and “ggplot2” packages in the R software.

### Analysis of gut microbiota

The gut microflora databases, including Disbiome, HMDAD, gutMEGA, and gutMDisorder, retrieved the T1DM and DN-related gut bacteria, among which the intersection was taken using the jvenn. The expression trends of gut bacteria in diabetes and nephropathy were analyzed using the GMrepo database.

### Quantification of short-chain fatty acids

The intestinal contents of mice were homogenized using a Precellys homogenizer (Bertin Technologies, Montigny Le Bretonneux, France) with 1.4 mm ceramic beads. Samples (10–30 mg) were homogenized twice in 1 mL of 70% isopropanol, with each round lasting 15 s of agitation followed by 60 s of rest. Subsequently, 300 μL of the homogenate was taken to determine the dry weight (DW) after overnight drying in a vacuum centrifuge. Fecal homogenates were diluted to 2.0 mg DW/mL, and samples were stored at −80°C, with precautions taken to keep them cold during processing.

For short-chain fatty acids (SCFAs) analysis, the supernatant obtained after centrifugation of the homogenate was derivatized. A 50 μL aliquot of the supernatant (2 mg DW/mL) was mixed with 50 μL of a standard solution containing 100 μg/mL of [13C, D3]-acetic acid, [D5]-propionic acid, and 500 μg/mL of [D7]-butyric acid, followed by the addition of 20 μL of 200 mM 3-nitrophenylhydrazine in 3% HCl and 20 μL of 120 mM N-(3-dimethylaminopropyl)-N′-ethylcarbodiimide, and incubated at 40°C for 30 min. The reaction was quenched with 200 μL of 0.1% formic acid for subsequent LC–MS/MS analysis.

Quantitative analysis of SCFAs was performed using liquid chromatography–tandem mass spectrometry (LC–MS/MS). A 1200 series binary pump, isocratic pump, and degasser (Agilent) were coupled to an API 4000 Q-Trap mass spectrometer (equipped with Turbo V ion spray, Applied Biosystems) through an HTC Pal autosampler (CTC Analytics). A Kinetex® 2.6 μm XB-C18, 50 × 2.1 mm column (Phenomenex, Torrance) was used with water containing 0.1% formic acid as mobile phase A and acetonitrile as mobile phase B. The gradient elution started at 10% B, increased to 20% B at 0.3 min, further increased to 23% B at 2.5 min, was held at 100% B from 2.6 to 3.0 min, and then re-equilibrated at 90% A from 3.1 to 4 min. The flow rate was set at 500 μL/min, column temperature at 60°C, and sample injection volume at 2 μL. To reduce contamination of the mass spectrometer, the column effluent was directed into the mass spectrometer from 55 to 160 s, with methanol delivered at 250 μL/min for the remainder of the run. Mass spectrometry was conducted in negative ion mode, with an ion spray voltage of −4,500 V, source heater temperature at 450°C, curtain gas at 25 psi, gas 1 at 55 psi, and gas 2 at 50 psi. Multiple reaction monitoring (MRM) mode was used for monitoring analytes, operating quadrupoles Q1 and Q3 at unit resolution (Liebisch et al., 2019).

### Flow cytometric sorting

Peripheral mouse blood monocytes were isolated using density-gradient centrifugation and washed three times with Roswell Park Memorial Institute (RPMI) 1,640 medium (Gibco, Grand Island, NY). Peripheral blood monocytes were resuspended in 100 μL of 0.01 M PBS (1 × 10^7^ cells/well). The mouse kidney tissue was homogenized and digested to prepare a cell suspension. The kidney cell suspension was then centrifuged to obtain the cellular pellet of kidney tissue for subsequent flow cytometry analysis. CD4^+^ T reg cells were evaluated by flow cytometry. The surface markers were stained with phycocyanin (APC)-labeled anti-CD4 (1:100, 561,091, BD Biosciences, Franklin Lakes, NJ) or fluorescein isothiocyanate (FITC)-labeled anti-CD25 antibody (1:100, 555,431, BD Biosciences), and further treated with a BD fixation/transmission kit (BD Biosciences). Then, the analysis was performed using a FACS Calibur flow cytometer equipped with an argon laser. The acquisition was analyzed using the Cell Quest software (BD Biosciences).

### Statistical analysis

Data was analyzed using the SPSS 21.0 software (IBM, Armonk, NY). All quantitative data are presented as mean ± standard deviation. The normality and homogeneity of variance were conducted. The data conforming to normal distribution and homogeneous variance between two groups were analyzed by unpaired *t*-test, and the data among multiple groups were analyzed by one-way analysis of variance (ANOVA) or repeated measures ANOVA, followed by Tukey’s *post hoc* test. *p* < 0.05 was considered statistically significant.

## Results

### hUCMSCs-Exo@Ex-4 reduces blood glucose and proteinuria production in DN mice

**Figure 1:**
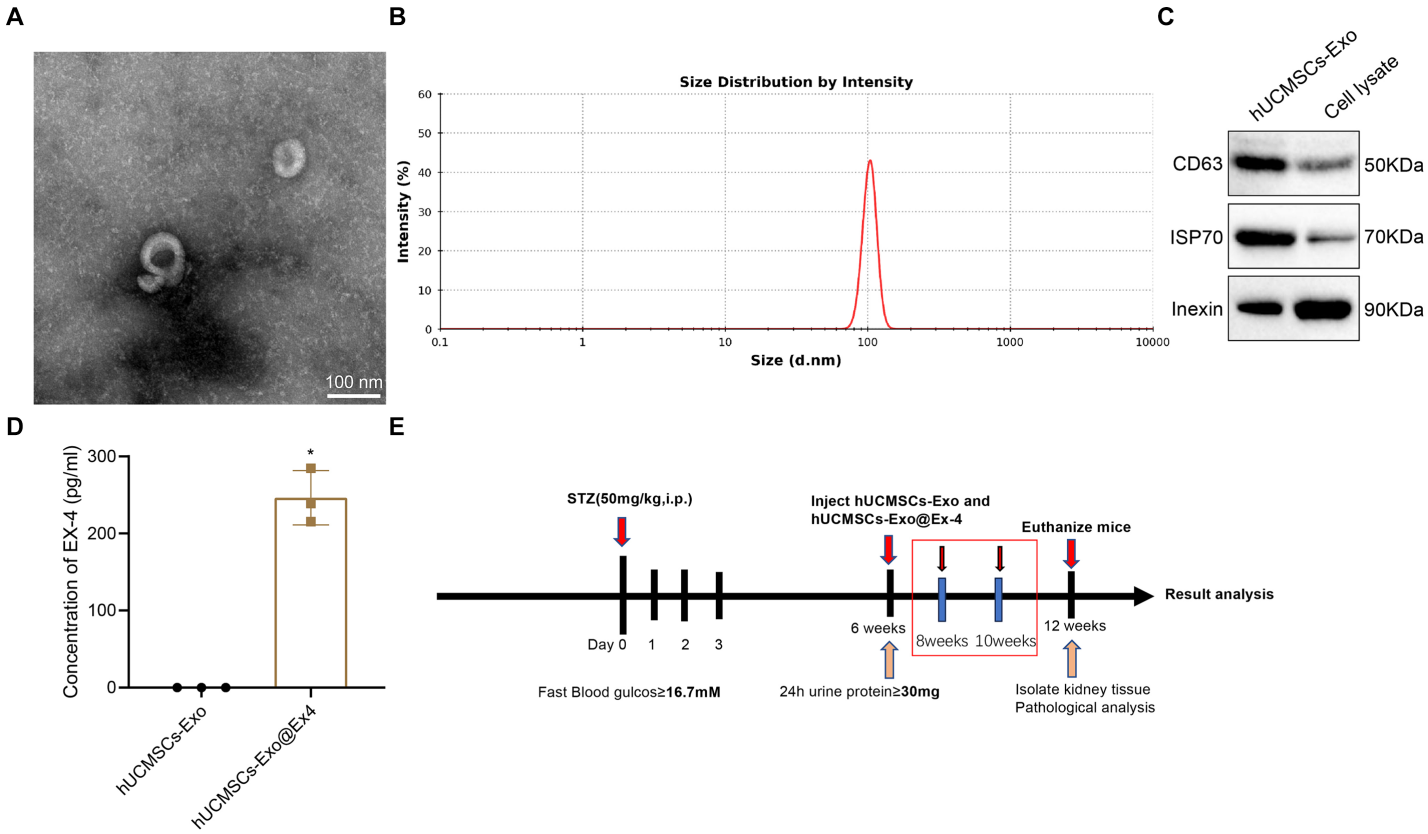
Isolation and identification of hUCMSCs-Exo and the Timeline of animal models. **(A)** Morphology of hUCMSCs-Exo under the TEM. **(B)** The Particle diameter of hUCMSCs-Exo detected by DLS. **(C)** CD63, HSP70, and Calnexin expression in hUCMSCs-Exo detected by Western blot analysis. **(D)** Ex-4 content in hUCMSCs-Exo and h UCMSCs-Exo@Ex-4 measured by ELISA. **(E)** Timeline of animal experiments. *p < 0.05 vs. hUCMSCs-Exo.

At first, exosomes were isolated from hUCMSCs, which TEM, DLS, and Western blot analysis characterized. The observations indicated that the isolated hUCMSCs-Exo was shaped like a saucer with a double membrane structure with a 30 and 100 nm diameter. The CD63 and HSP70 were highly expressed, and Calnexin was not expressed (Figures 1A–C). Ex-4 was loaded onto exosomes using electroporation, and ELISA displayed that the Ex-4 content was increased in hUCMSCs-Exo@Ex-4 (Figure 1D).

**Figure 2:**
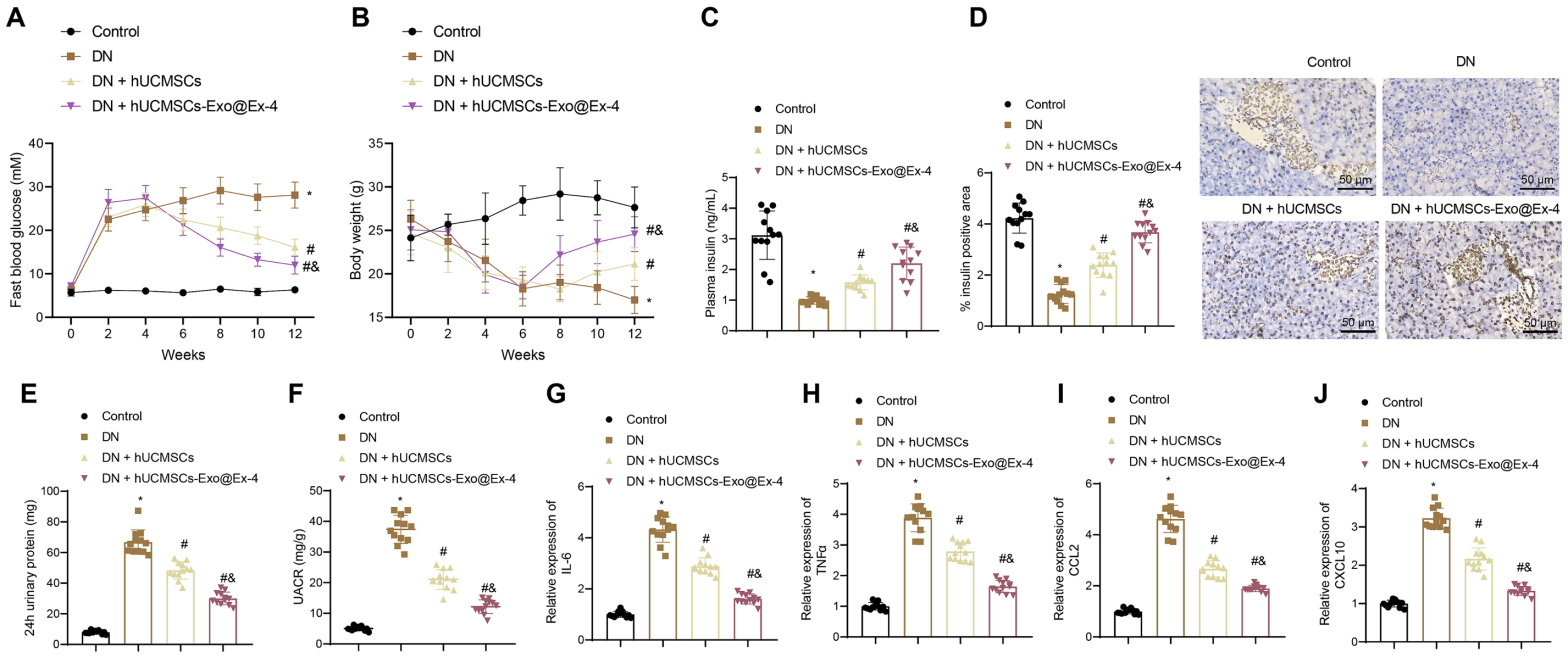
hUCMSCs-Exo@Ex-4 affects blood glucose production and proteinuria in DN mice. DN mice were injected with hUCMSCs-Exo or hUCMSCs-Exo@Ex-4 (*n* = 12). **(A)** Blood glucose in DN mice was detected once every 2 weeks. **(B)** Body weight of DN mice detected once every 2 weeks. **(C)** The Plasma insulin level in DN mice after 12 weeks of STZ injection. **(D)** Pancreatic tissue sections of DN mice stained by immunohistochemistry with insulin antibody, with the percentage of stained areas quantified. **(E)** Urinary protein level of DN mice. **(F)** UACR of DN mice. **(G–J)** expression of inflammatory cytokines IL-6 **(G)** and TNFα **(H)** and chemokines CCL2 **(I)** and CXCL10 **(J)** in the renal tissues of DN mice measured by RT-qPCR. *p < 0.05 vs. control mice; ^#^*p* < 0.05 vs. DN mice; and *p* < 0.05 vs. DN mice injected with hUCMSCs-Exo.

The STZ-induced DN mouse model was then constructed. The Timeline of the animal model is shown in Figure 1E. After STZ injection, all the injected mice showed increased blood glucose (Figure 2A), weight loss (Figure 2B), and reduced plasma insulin level (Figure 2C). The DN mice injected with hUCMSCs-Exo or hUCMSCs-Exo@Ex-4 presented decreased blood glucose and elevated body weight and plasma insulin level (Figures 2A–C).

Immunohistochemical staining displayed that the pancreatic islet cells were reduced in the DN mice, while the number of pancreatic islet cells was increased after injection with hUCMSCs-Exo or hUCMSCs-Exo@Ex-4 (Figure 2D).

Moreover, as shown in Figures 2E,F, urinary protein and UACR were increased at 24 h after 12 weeks of STZ injection in DN mice, while urinary protein and UACR were reduced at 24 h after injection with hUCMSCs-Exo or hUCMSCs-Exo-Exo@Ex-4. It was also found that the expression of inflammatory cytokines IL-6 and TNFα and chemokines CCL2 and CXCL10 was increased in the renal tissues of DN mice, while expression of these factors was downregulated after injection with hUCMSCs-Exo or hUCMSCs-Exo@Ex-4 (Figures 2G–J).

Moreover, the above experiments showed that hUCMSCs-Exo@Ex-4 had a better therapeutic effect than hUCMSCs-Exo injection alone. Thus, it can be speculated that hUCMSCs-Exo carrying Ex-4 decreased blood glucose and the production of proteinuria in DN mice.

### hUCMSCs-Exo@Ex-4 ameliorates kidney injury in DN mice

It is known that increased blood glucose leads to renal hypertrophy and glomerulosclerosis. Thus, we further explored whether hUCMSCs-Exo@Ex-4 could attenuate kidney injury in DN mice.

**Figure 3:**
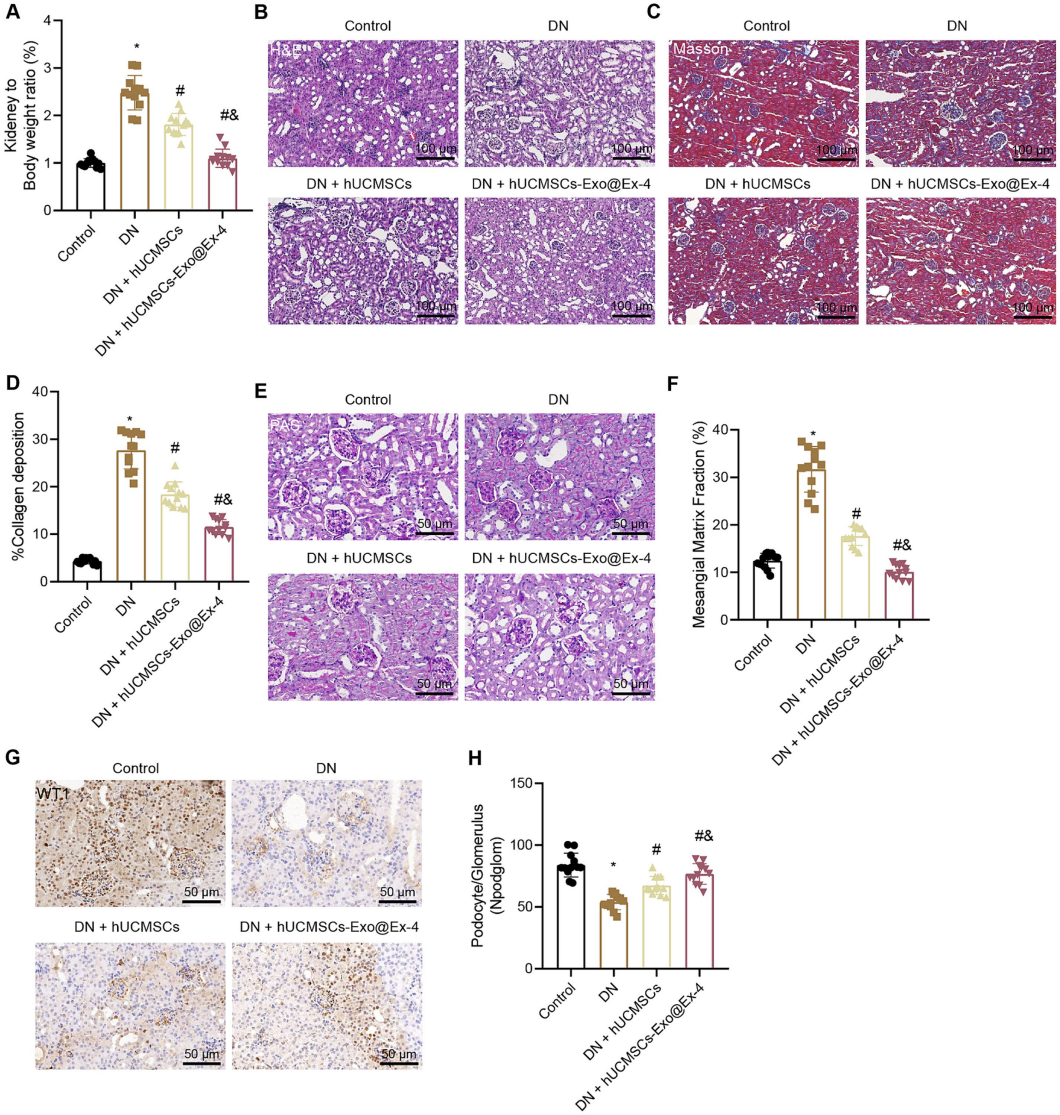
Effects of hUCMSCs-Exo@Ex-4 on kidney injury in DN mice. DN mice were injected with hUCMSCs-Exo or hUCMSCs-Exo@Ex-4 (*n* = 12). **(A)** Ratio of kidney tissue weight to body weight of DN mice. **(B)** Kidney histopathology of DN mice detected by H&E staining. **(C,D)** Images **(C)** and quantitation **(D)** showing renal fibrosis of DN mice detected by Masson trichrome staining. **(E,F)** Images **(E)** and quantitation **(F)** showing glomerular basement membrane thickness in DN mice detected by PAS staining. **(G,H)** Images **(G)** and quantitation **(H)** showing podocytes in the glomeruli of the DN mice were quantified by measuring WT-1 expression using immunohistochemistry. *p < 0.05 vs. control mice; ^#^*p* < 0.05 vs. DN mice; and *p* < 0.05 vs. DN mice injected with hUCMSCs-Exo.

The observations showed that the kidney-to-mouse weight ratio was increased in DN mice, while injections of hUCMSCs-Exo and hUCMSCs-Exo@Ex-4 prevented kidney hypertrophy. In the 12^th^ week, the kidney-to-mouse weight ratio displayed no difference compared with the control mice (Figure 3A). H&E staining, Masson trichrome staining, and PAS staining showed inflammatory cell infiltration, granular degeneration in tubular interstitial tissues, glomerular and interstitial fibrosis, and thickened glomerular basement membrane in DN mice, while these symptoms were alleviated after injection with hUCMSCs-Exo and hUCMSCs-Exo@Ex-4 (Figures 3B–F).

Podocytes, acting as a structural component of the glomerular filtration barrier, play an important role in the development of DN. Thus, the podocytes were quantified in the glomeruli of DN mice by observing the WT-1 expression using immunohistochemistry, which presented that the number of podocytes was reduced in the glomeruli of the DN mice, while it was increased in DN mice injected with hUCMSCs-Exo or hUCMSCs-Exo@Ex-4 (Figures 3G,H). The therapeutic effects of hUCMSCs-Exo@Ex-4 were better than that of hUCMSCs-Exo.

These findings indicated that hUCMSCs-Exo@Ex-4 could relieve renal hypertrophy, glomerular injury, reduced podocytes, and renal fibrosis in glomeruli in DN mice.

### Infiltration of nTreg cells is reduced in DN

**Figure 4:**
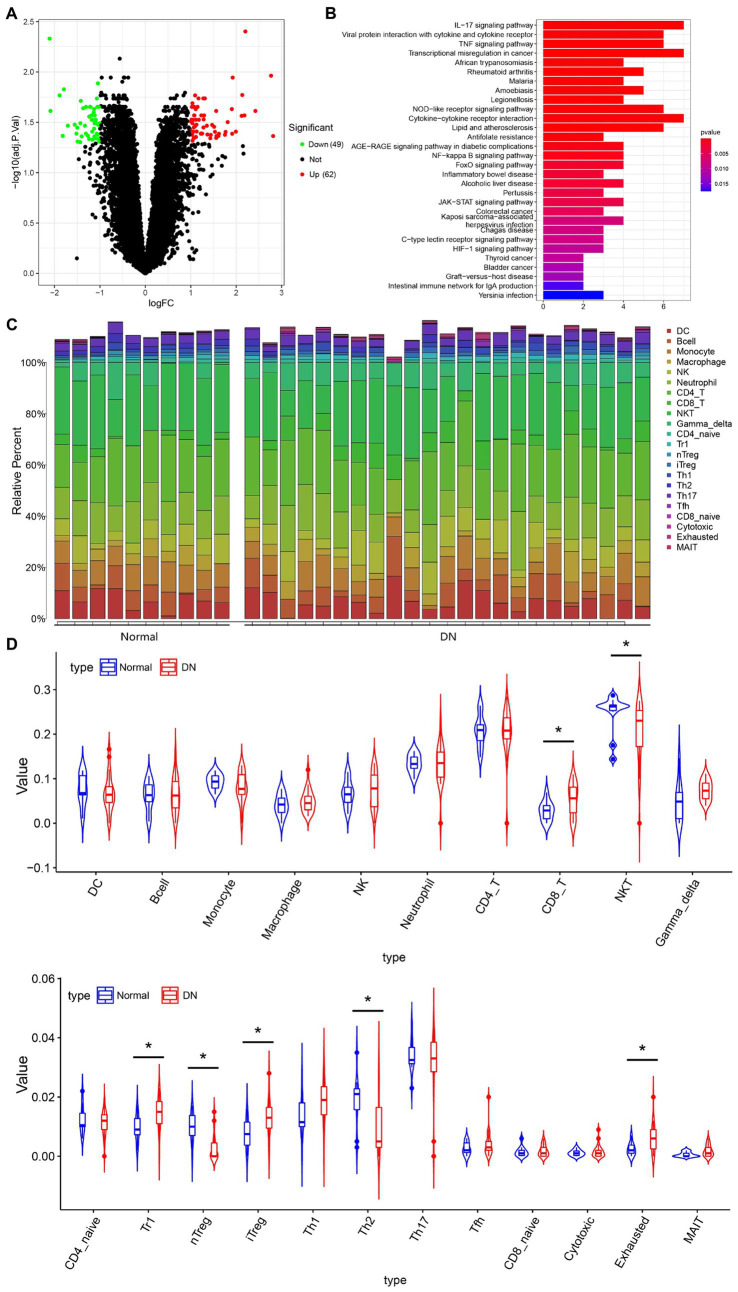
The immune cells that mainly function in DN based on immune-infiltration analysis. **(A)** Differentially expressed genes in microarray data GEO142153 (including 10 normal and 23 DN samples) and the volcano map. Green represents significantly downregulated genes, red represents significantly upregulated genes, and black represents the genes without differential expression. **(B)** KEGG enrichment pathway analysis of upregulated genes. **(C)** Heat map of the infiltration score of 22 immune cells. **(D)** Violin plot of the infiltration score of 22 immune cells in peripheral blood of healthy individuals and DN patients. *p < 0.05.

To explore whether hUCMSCs-Exo@Ex-4 improves kidney injury in DN through the immune pathway, the DN-related microarray data GSE142153 (including 10 normal and 23 DN samples) was downloaded from the GEO database. Forty-nine downregulated genes and 62 upregulated genes were obtained through differential analysis using the R language “Limma” package (Figure 4A; Supplementary Figure S1). KEGG enrichment analysis revealed that the upregulated genes were mainly enriched in the IL-17 signaling pathway, the transcriptional dysregulation in cancer, and the cytokine-cytokine receptor interaction pathways (Figure 4B).

The immuCellAI method was used to assess the differences in immune infiltration between normal samples and DN samples, which indicated that the infiltration of CD8^+^ T cells, Tr1 cells, iTreg cells, and exhausted T cells was increased, while the infiltration of NKT cells, natural regulatory T cells (Treg) cells, and Th2 cells were decreased in DN samples (Figures 4C,D). Since IL-17 is secreted by CD4^+^ T cells, Treg cells can effectively inhibit the dysfunction of effector T cells, which can effectively suppress the production of IL-17 to avoid the inflammatory response. As shown in Figure 4D, the immune infiltration of nTreg cells was reduced in DN.

Thus, we hypothesized that the DN kidney injury improved by hUCMSCs-Exo@Ex-4 might be related to nTreg cells.

### hUCMSCs-Exo@Ex-4 induces CD4^+^ Treg cells to attenuate kidney injury in DN mice

**Figure 5:**
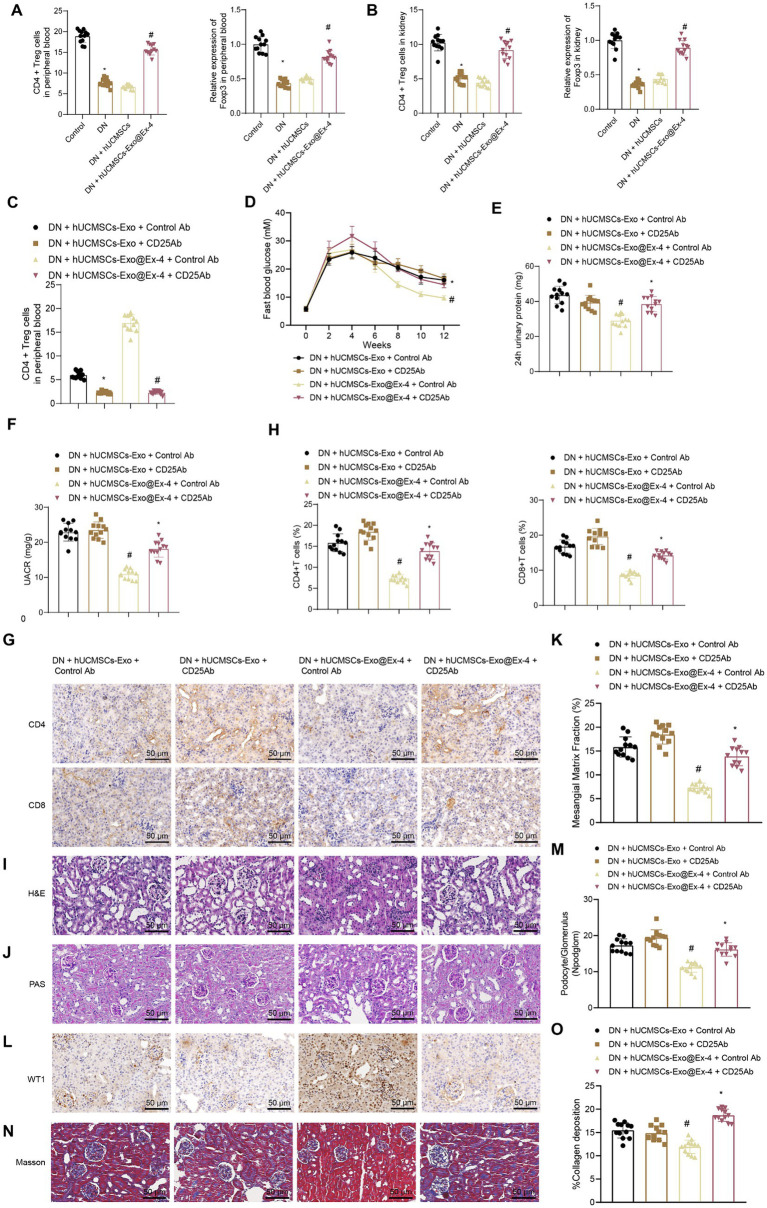
Effects of hUCMSCs-Exo@Ex-4 on kidney injury in DN mice by inducing CD4^+^ Treg cells. **(A)** Flow cytometry to detect CD4+ Treg cells in peripheral blood of each group of mice, and qRT-PCR to measure the expression levels of Foxp3. **(B)** Flow cytometry to detect CD4^+^ Treg cells in kidney tissues of each group of mice, and qRT-PCR to measure the expression levels of Foxp3. **(C)** CD4^+^ Treg cells in the peripheral blood of DN mice detected by flow cytometry. **(D)** Blood glucose of DN mice detected once every 2 weeks. **(E)** Urinary protein level of DN mice. **(F)** UACR of DN mice. **(G,H)**, Images **(G)** and quantitation **(H)** showing infiltration of CD4^+^ T cells and CD8^+^ T cells in DN mice detected by immunohistochemistry. **(I)** Kidney histopathology of DN mice detected by H&E staining. **(J,K)** Images **(J)** and quantitation **(K)** showing renal fibrosis of DN mice detected by Masson trichrome staining. **(L,M)** Images **(L)** and quantitation **(M)** showing podocytes in the glomeruli of the DN mice were quantified by measuring WT-1 expression using immunohistochemistry. **(N,O)** Images **(N)** and quantitation **(O)** showing glomerular basement membrane thickness in DN mice detected by PAS staining. *p < 0.05 vs. DN mice injected with hUCMSCs-Exo@Ex-4 + control Ab or Control mice, and ^#^*p* < 0.05 vs. DN mice injected with hUCMSCs-Exo + control Ab or DN mice injected with hUCMSCs-Exo.

It has attracted great attention that CD4^+^CD25^+^FoxP3^+^ natural regulatory T cells (Treg) act as an effective immunosuppressive population in inflammatory diseases. Therefore, we hypothesized that hUCMSCs-Exo@Ex-4 may improve DN kidney injury by inducing CD4^+^ Treg. The peripheral blood and kidney tissue samples of the mice in each group were collected, and CD4^+^ Treg cells were examined using flow cytometry, which indicated that the number of CD4^+^ Treg cells and Foxp3 expressions decreased in the DN mice. There was no significant difference in the number of CD4^+^ Treg cells and Foxp3 expression between DN mice and DN mice injected with hUCMSCs-Exo. However, CD4^+^ Treg cells and Foxp3 expression were elevated in the DN mice injected with hUCMSCs-Exo@Ex-4 (Figures 5A,B).

CD4^+^ Treg cells were exhausted by anti-CD25 antibody (Figure 5C) and found that anti-CD25 antibody reversed the therapeutic effects of hUCMSCs-Exo@Ex-4 in DN mice but had little impact on the effects of hUCMSCs-Exo. Anti-CD25 antibody combined with hUCMSCs-Exo@Ex-4 increased blood glucose (Figure 5D), urinary protein (Figure 5E), and UACR of DN mice (Figure 5F) compared with DN mice injected with hUCMSCs-Exo@Ex-4 alone.

Immunohistochemistry showed that compared with DN mice injected with hUCMSCs-Exo@Ex-4, infiltration of CD4^+^ T cells and CD8^+^ T cells was increased in DN mice injected with anti-CD25 antibody and hUCMSCs-Exo@Ex-4, while no obvious difference was found when compared with the DN mice injected with hUCMSCs-Exo (Figures 5G,H). H&E staining (Figure 5I), PAS staining (Figures 5J,K), immunohistochemistry (Figures 5L,M), and Masson trichrome staining (Figures 5N,O) indicated that CD4^+^ Treg depletion negated the therapeutic effects of hUCMSCs-Exo@Ex-4 in glomerular injury, reduced podocytes, and renal fibrosis in DN mice, but had little impact on the effects of hUCMSCs-Exo.

These findings confirmed that hUCMSCs-Exo@Ex-4 relieved kidney injury by inducing CD4^+^ Treg cells in DN mice.

### Induction of CD4^+^ Treg cells is correlated with the abundance of *Prevotella* in DN mice

**Figure 6:**
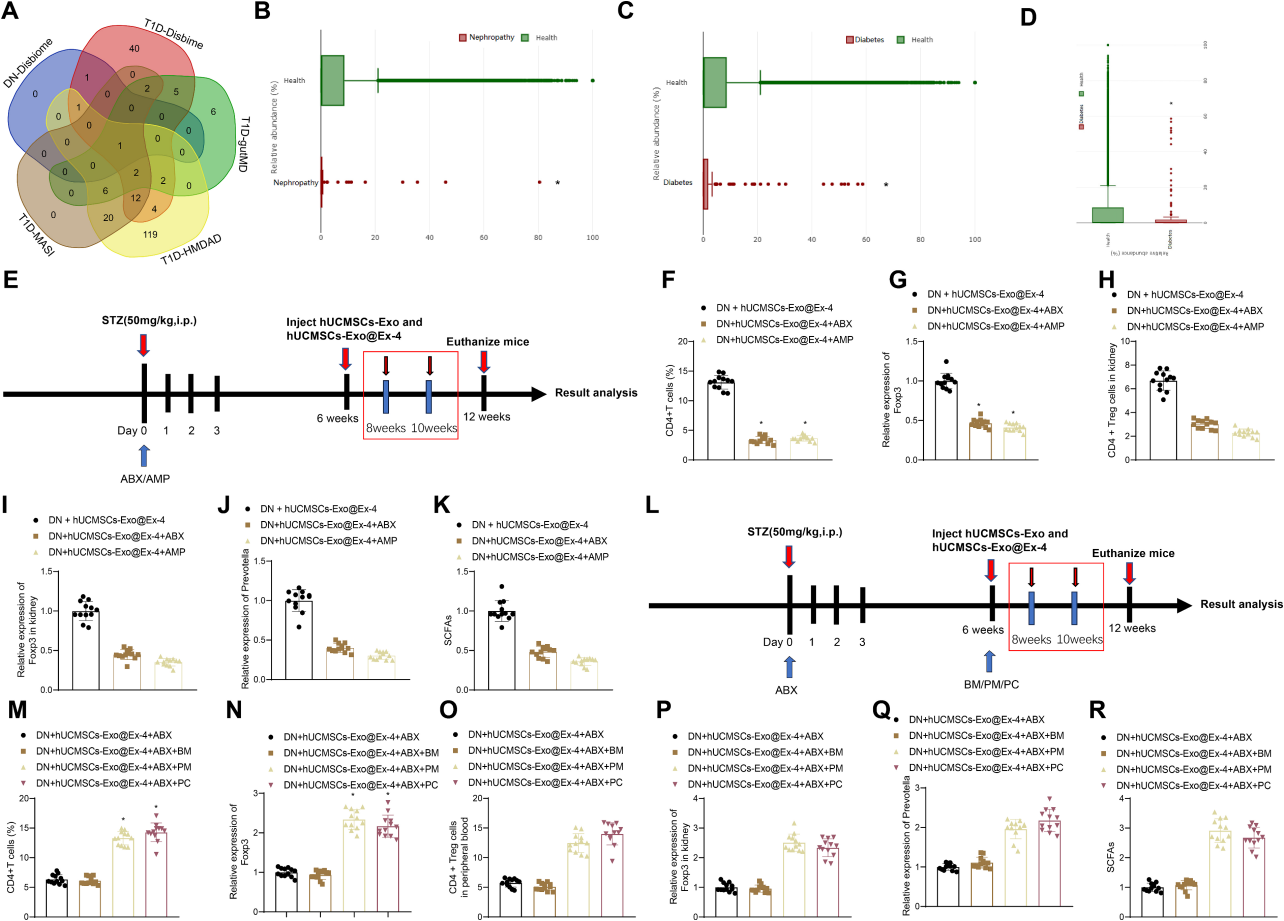
*Prevotella* affects the induction of CD4^+^ Treg cells by hUCMSCs-Exo@Ex-4. **(A)** Veen diagram of intersection between T1D-related and DN-related gut microbiotas in Disbiome, gutMDisorder, HMDAD, MASI database. **(B)** The number of *Prevotella* in healthy individuals and kidney patients based on the GMrepo database (*n* = 51. This taxon was found in 51 samples/runs of current phenotype collected in the database.). **(C)** The number of *Prevotella* in healthy people and patients with diabetes based on the GMrepo database (*n* = 120. This taxon was found in 51 samples/runs of current phenotype collected in the database). **(D)** PCR detection of changes in gut *Prevotella* microbiota; **(E,L)** Schematic diagram of hUCMSCs-Exo@Ex-4 combined with antibiotic treatment; **(F,M)** Flow cytometry analysis of CD4 + Treg cells in peripheral blood of each group of mice; **(G,N)** qRT-PCR detection of Foxp3 expression levels in peripheral blood; **(H,O)** Flow cytometry analysis of CD4 levels in mouse kidney tissue; **(I,P)** qRT-PCR detection of Foxp3 expression levels in kidney tissue; **(J,Q)** qRT-PCR detection of *Prevotella* levels; **(K,R)** Liquid chromatography quantification of SCFAs levels. Each group contains 12 mice, * indicates *p* < 0.05 compared to DN + hUCMSCs-Exo@Ex-4 group/DN + hUCMSCs-Exo@Ex-4 + ABX + BM group.

A growing number of studies show that gut microbiota plays a vital role in the development of DN. The type 1 diabetes (T1D)-related gut microbiotas were obtained through Disbiome (Supplementary Table S2), HMDAD (Supplementary Table S3), gutMDisorder (Supplementary Table S4), and MASI (Supplementary Table S5) database, and DN-related gut microbiotas were found from the Disbiome database (Supplementary Table S6). These gut microbiotas in several databases were intersected, and only gut microbiota *Prevotella* was obtained (Figure 6A), and its abundance in T1DM and DN was reduced. GMrepo database indicated that the number of *Prevotella* decreased in patients with kidney disease or diabetes compared with healthy individuals (Figures 6B,C). It is reported that *Prevotella*, a bacterium that mainly produces SCFAs, is a known producer of anti-inflammatory metabolites, which can reduce Th17 polarization and promote the differentiation of anti-inflammatory Treg/Tr1 cells in the intestine. We further validated the analysis results from the database by PCR testing fecal samples of DN model mice (Figure 6D). Our findings revealed a significant reduction in the Prevotella microbiota in the fecal samples of DN mice compared to those of normal mice, suggesting that Prevotella plays a significant role in the gut dysbiosis of DN mice.

We moved to investigate whether *Prevotella* is involved in the induction of CD4^+^ Treg cells by hUCMSCs-Exo@Ex-4. Before injection with hUCMSCs-Exo@Ex-4, DN mice were treated with ABX or AMP to disrupt the gut microbiota (Figure 6E). The results reveal that compared to the treatment group receiving hUCMSCs-Exo@Ex-4 alone, the administration of oral ABX or AMP significantly reduced the quantity of CD4^+^Treg cells in peripheral blood and kidney tissues. Moreover, there was a decrease in the expression of Foxp3 (Figures 6F–I). Additionally, the presence of Prevotella in the gut microbiota significantly decreased, and the fecal content of short-chain fatty acids (SCFAs) also declined (Figures 6J,K).

To further validate the induction of CD4 + Treg cells depending on Prevotella, after ABX treatment, we orally administered two different Prevotella strains to the mice, namely *P. melaninogenica* (PM) and *P. copri* (PC) (Figure 6L). The results showed that compared to the group receiving oral gavage of blank medium (BM), the quantities of CD4 + Treg cells in peripheral blood and kidney tissues significantly increased in the PM and PC groups, along with increased Foxp3 expression (Figures 6M–P). Furthermore, following the treatment with PM or PC, the proportion of Prevotella in the gut microbiota was restored, and the content of SCFAs in feces also increased (Figures 6Q,R).

The obtained data suggested that the induction of CD4^+^ Treg cells was related to the abundance of *Prevotella* in DN mice.

### hUCMSCs-Exo@Ex-4 ameliorates kidney injury in DN mice by regulating gut microbiota metabolism

The above findings have shown that the induction of CD4^+^ Treg cells by hUCMSCs-Exo@Ex-4 was associated with *Prevotella*. Therefore, we further explored the effects of hUCMSCs-Exo@Ex-4 inducing CD4^+^ Treg cells on kidney injury in DN mice by affecting gut microbiota metabolism.

**Figure 7:**
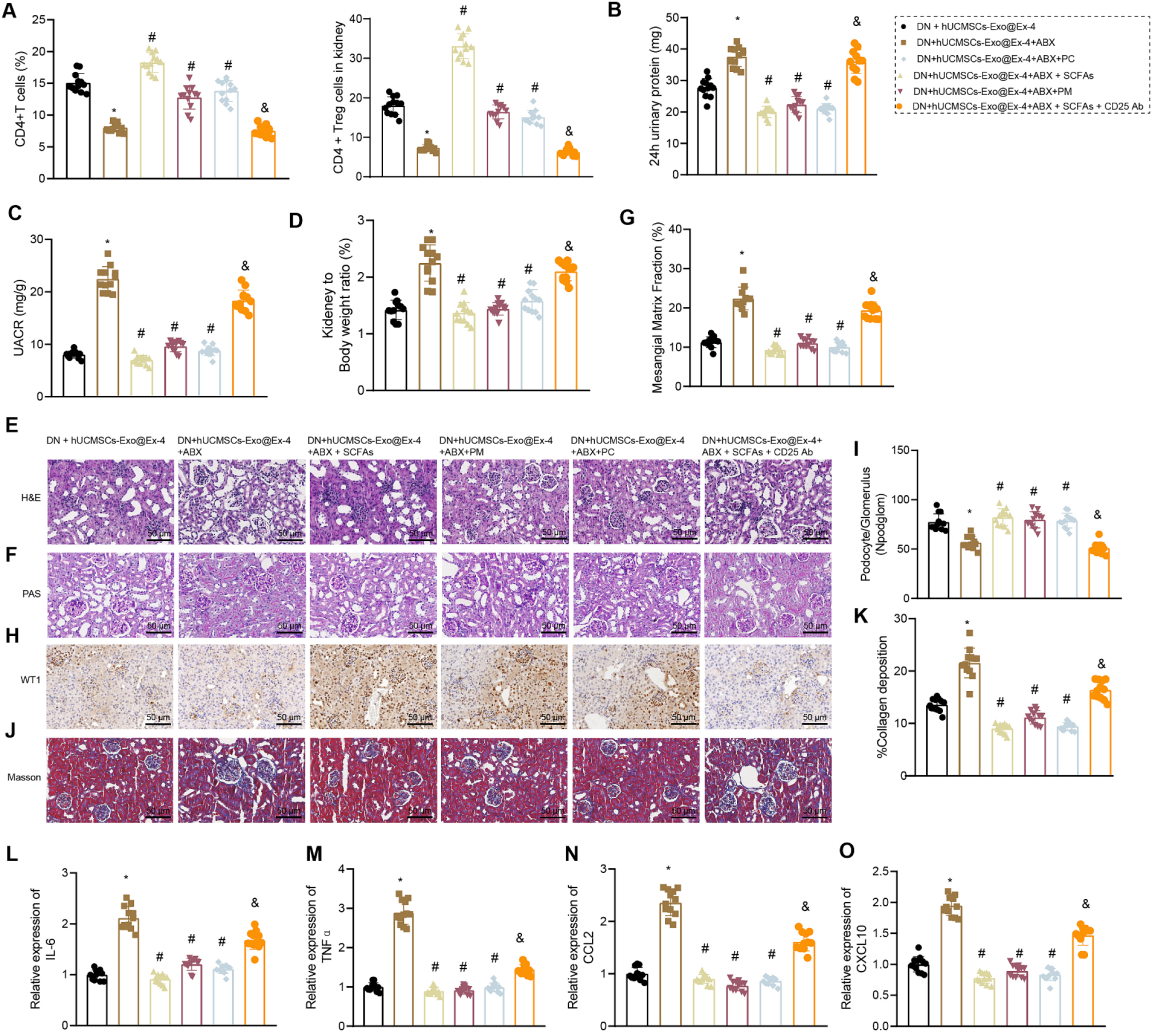
Effects of hUCMSCs-Exo@Ex-4 and the induced CD4^+^ Treg cells on kidney injury in DN mice via gut microbiota metabolism. DN mice were injected with hUCMSCs-Exo@Ex-4 + ABX or combined with SCFAs, PM, or PC, followed by anti-CD25 antibody treatment (*n* = 12). **(A)** CD4^+^ Treg cells in the peripheral blood of DN mice detected by flow cytometry. **(B)** Urinary protein level of DN mice at 24 h. **(C)** UACR of DN mice. **(C)** Measurement of the urine albumin/creatinine ratio (UACR) in each group of mice. **(D)** Ratio of kidney tissue weight to body weight of DN mice. **(E)** Kidney histopathology of DN mice detected by H&E staining. **(F,G)** Images **(F)** and quantitation **(G)** showing glomerular basement membrane thickness in DN mice detected by PAS staining. **(H,I)** Images **(H)** and quantitation **(I)** showing podocytes in the glomeruli of the DN mice were quantified by measuring WT-1 expression using immunohistochemistry. **(J,K)** Images **(J)** and quantitation **(K)** showing renal fibrosis of DN mice detected by Masson trichrome staining. **(L–O)**, expression of inflammatory cytokines IL-6 **(L)** and TNFα **(M)** and chemokines CCL2 **(N)** and CXCL10 **(O)** in the renal tissues of DN mice measured by RT-qPCR. *p < 0.05 vs. DN mice injected with hUCMSCs-Exo@Ex-4; ^#^*p* < 0.05 vs. DN mice injected with hUCMSCs-Exo@Ex-4 + ABX; ^#^*p* < 0.05 vs. DN mice injected with hUCMSCs-Exo@EX-4 + ABX + SCFAs.

Firstly, using flow cytometry to analyze the quantity of CD4^+^ Treg cells in each group of mice, the results demonstrated the following: compared to the DN + hUCMSCs-Exo@Ex-4 group, a reduction in the number of CD4^+^ Treg cells in peripheral blood and kidney tissues was evident in the DN + hUCMSCs-Exo@Ex-4 + ABX group. Conversely, administration of SCFAs/PM/PC via gavage significantly increased the CD4^+^ Treg cell count. Furthermore, the use of anti-CD25 antibodies effectively reduced the number of CD4+ Treg cells (Figure 7A).

It was also found that that urinary protein, UACR, and the ratio of kidney to mouse weight were elevated in DN mice injected with hUCMSCs-Exo@Ex-4 + ABX, which were abolished by further treatment with SCFAs/PM/PC (Figures 7B–D). H&E staining (Figure 7E), PAS staining (Figures 7F,G), immunohistochemistry (Figures 7H,I), and Masson trichrome staining (Figures 5J,K) indicated that more inflammatory cell infiltration, thickened glomerular basement membrane, reduced number of podocytes, and increased glomerular interstitial fibrosis in DN mice injected with hUCMSCs-Exo@Ex-4 + ABX, while further treatment with SCFAs/PM/PC effectively improved the above symptoms and played a protective role in kidney injury in DN.

In addition, RT-qPCR indicated that expression of inflammatory cytokines IL-6 and TNFα and chemokines CCL2 and CXCL10 was increased in the renal tissues of DN mice injected with hUCMSCs-Exo@Ex-4 + ABX, while expression of these factors was reduced after further treatment with SCFAs/PM/PC (Figures 7L–O). Moreover, anti-CD25 antibodies could effectively reverse the treatment effect with SCFAs, indicating that the lessened effect of SCFAs on DN kidney injury depended on CD4^+^ Treg cells (Figures 7B–O).

Thus, it could be concluded that hUCMSCs-Exo@Ex-4 induced CD4^+^ Treg cells to attenuate kidney injury in DN mice by regulating gut microbiota metabolism.

## Discussion

DN is characterized by glomerular hypertrophy, glomerular basement membrane thickening, glomerulosclerosis, and tubulointerstitial fibrosis (Rayego-Mateos et al., 2020). Inflammation is also implicated in the progression of DN. Accumulated evidence has demonstrated that MSCs-derived exosomes may serve as a potential therapeutic strategy for DN due to their capability to curb fibrosis and inflammation (Ebrahim et al., 2018). In the current work, we confirmed that hUCMSCs-Exo@Ex-4 relieved kidney injury in DN mice by inducing CD4^+^ Treg cells, which was related to the abundance of *Prevotella*.

The obtained results indicated that hUCMSCs-Exo@Ex-4 reduced blood glucose and proteinuria production to effectively ameliorate kidney injury in DN mice, as manifested by attenuation of glomerular and interstitial fibrosis and thickened glomerular basement membrane and decreased expression of inflammatory cytokines IL-6 and TNFα and chemokines CCL2 and CXCL10. Proteinuria is the clinical hallmark of diabetic kidney disease. Chemokine monocyte chemoattractant protein 1 (MCP1), also known as CCL2, is highly expressed in the glomerular and renal tubular epithelium in diabetic patients. hUCMSCs could prevent the progression of DN by repressing inflammation and fibrosis (Xiang et al., 2020). MSC-derived exosomes reduce levels of blood glucose, serum creatinine, 24-h urinary protein, and kidney weight/body weight to inhibit kidney fibrosis in DN rats (Hao et al., 2021), which is consistent with our findings. Moreover, a prior study has proved that Ex-4 attenuates renal tubular injury by inhibiting inflammation in STZ-induced diabetic mice. Another study has also confirmed that Ex-4 could ameliorate the pathological processes of DN. These findings supported that hUCMSCs-Exo@Ex-4 could effectively attenuate kidney injury in DN mice.

Moreover, the obtained data revealed that infiltration of nTreg cells was reduced in DN and hUCMSCs-Exo@Ex-4 induced CD4^+^ Treg cells to attenuate kidney injury in DN mice. It is known that immune cell infiltration is implicated in the development of DN. Treg cells could inhibit the progression of DN by exerting anti-inflammatory effects. Recent evidence suggests that Treg cells are able to relieve renal injury by inhibiting inflammation in the diabetic kidney, highlighting an important role for Treg cells in the pathogenesis of DN. Besides, induction of CD4^+^ Treg cells was correlated with the abundance of *Prevotella* in DN mice. Consistently, the relative abundance of *Prevotella* is positively correlated with the number of CD4^+^ Treg cells (Wang et al., 2022). We also demonstrated that hUCMSCs-Exo@Ex-4 regulates gut microbiota metabolism to attenuate kidney injury in DN mice. Evidence demonstrates that dysbiosis of gut microbiota has gradually become a highly potential therapeutic agent for DN. The genus *Prevotella* contains over 50 characterized species that are highly abundant in multiple body sites, which are critical regulators in the balance between health and disease. Gut microbiota modulated by resveratrol inhibit inflammation to protect against DN in mice. Our results indicated that hUCMSCs-Exo@Ex-4 could induce CD4^+^ Treg cells by regulating gut microbiota metabolism in DN and greatly help understand the role of hUCMSCs-Exo@Ex-4 in relieving kidney injury in DN.

Our study demonstrated that hUCMSCs-Exo carrying Ex-4 can effectively ameliorate kidney injury to prevent the progression of DN by inducing CD4^+^ Treg cells via gut microbiota metabolism. These findings provide a new mechanistic insight into the potential use of Ex-4 shuttled by hUCMSCs-Exo as a new therapeutic approach for DN. Further studies are required to shed light on the molecular mechanistic basis of hUCMSCs-Exo@Ex-4 in inducing CD4^+^ Treg cells via gut microbiota metabolism in kidney injury in DN.

## Data availability statement

The original contributions presented in the study are included in the article/Supplementary material, further inquiries can be directed to the corresponding authors.

## Ethics statement

The animal study was approved by the Animal Care and Use Committee of the Key Laboratory of Internal Medicine, Ministry of Education, and the Beijing Research Center of Dongzhimen Hospital, Beijing University of Chinese Medicine (approval no. 21–58). The study was conducted in accordance with the local legislation and institutional requirements.

## Author contributions

LW: Conceptualization, Data curation, Formal analysis, Resources, Writing – original draft. AL: Software, Supervision, Validation, Visualization, Writing – review & editing. JH: Funding acquisition, Investigation, Methodology, Project administration, Writing – review & editing.

## Footnotes

^1^Source: https://www.atcc.org/
