# Supplementary figures and images for: Exendin-4-enriched exosomes from hUCMSCs alleviate diabetic nephropathy via gut microbiota and immune modulation
Source: Front Microbiol. 2024 Aug 30;15:1399632. doi: 10.3389/fmicb.2024.1399632 (PMC11392743; doi:10.3389/fmicb.2024.1399632)

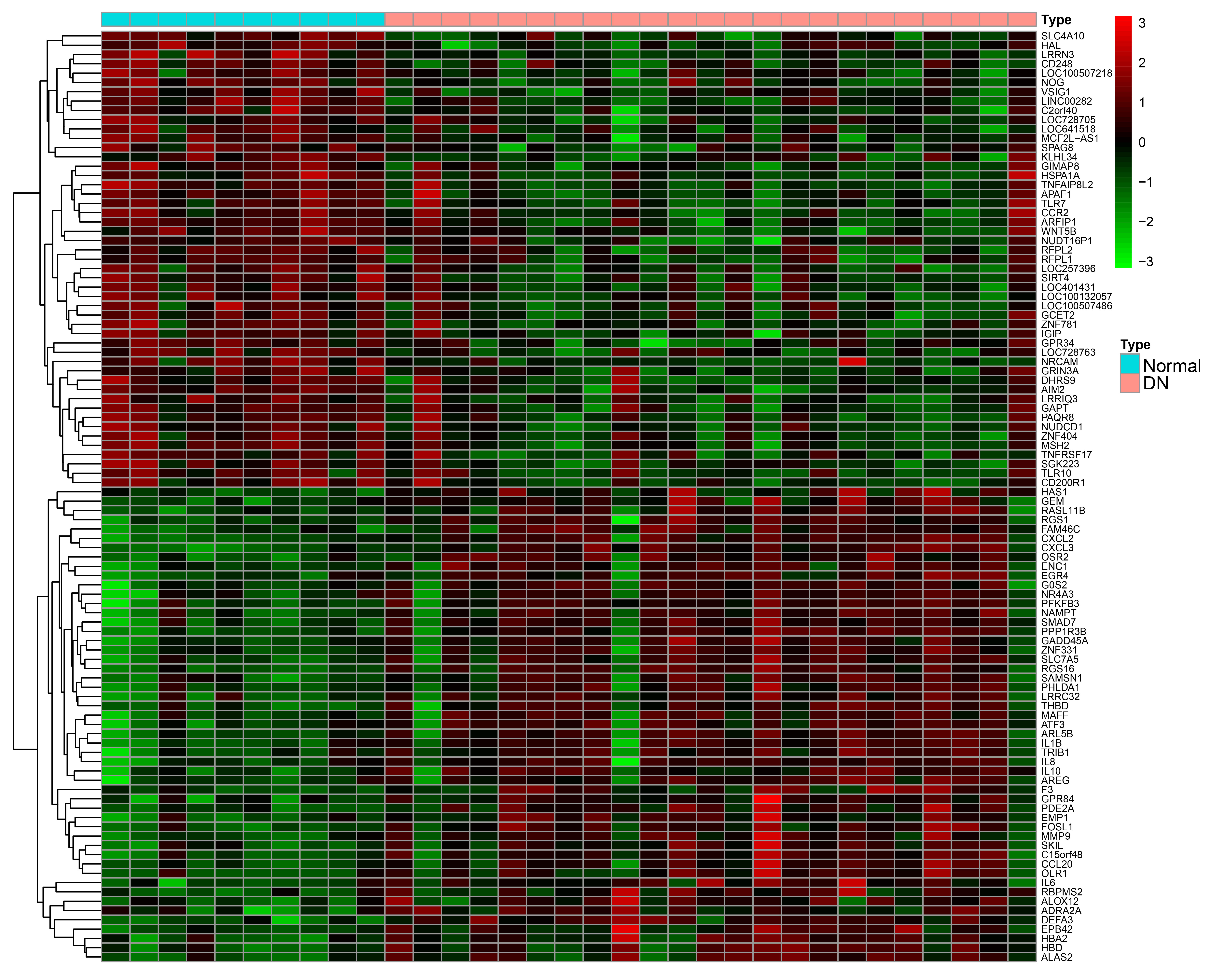

Supplement: Supplementary file 1 [file Image_1.JPEG]
