# Supplementary material for: Exendin-4-enriched exosomes from hUCMSCs alleviate diabetic nephropathy via gut microbiota and immune modulation
Source: Front Microbiol. 2024 Aug 30;15:1399632. doi: 10.3389/fmicb.2024.1399632 (PMC11392743; doi:10.3389/fmicb.2024.1399632)
Supplement: Supplementary file 2 [file Table_1.DOCX]

**Supplementary Table 1** Primer sequences of RT-qPCR

| Gene | Primer sequence（5'-3'） |
| --- | --- |
| GAPDH | F: CCCTTAAGAGGGATGCTGCC |
|  | R: TACGGCCAAATCCGTTCACA |
| IL-6 | F: TGATGGATGCTACCAAACTGGA |
|  | R: TGTGACTCCAGCTTATCTCTTGG |
| TNF-α | F: GATCGGTCCCCAAAGGGATG |
|  | R: CCACTTGGTGGTTTGTGAGTG |
| CCL2 | F: AACTGCATCTGCCCTAAGGT |
|  | R: AGGCATCACAGTCCGAGTCA |
| CXCL10 | F: TGCCGTCATTTTCTGCCTCA |
|  | R: AGGCTCGCAGGGATGATTTC |
| Foxp3 | F: GCGAAAGTGGCAGAGAGGTAT |
|  | R: AGCTTTCTTCTGTCTGGAGTGG |
| P. copri | F: CGCGAACTGGTTTCCTTGA  R: ACCGCTACACCACGAATTCC |
| P. melaninogenica | F: CGTCATGAAGGAGATTGG  R: ATAGAACCGTCAACGCTC |
| Universal bacterial 16s rRNA | F: CCATGAAGTCGGAATCGCTAG  R: GCTTGACGGGCGGTGT |

Note: RT-qPCR, reverse transcription quantitative polymerase chain reaction; F, forward; R, reverse; GAPDH, glyceraldehyde-3-phosphate dehydrogenase; IL-6, interleukin 6; TNF-α, tumor necrosis factor-α; CCL2, monocyte chemoattractant protein-1; CXCL10, C-X-C motif chemokine 10; Foxp3, Forkhead box P3.
